# From Intensification to Optimization: Balancing Efficacy, Safety, and Costs in High-Risk Localized Soft Tissue Sarcomas

**DOI:** 10.3390/cancers17101724

**Published:** 2025-05-21

**Authors:** Bruno Fuchs, Georg Schelling, Christoph Glanzmann, Gabriela Studer, on behalf of the Swiss Sarcoma Network

**Affiliations:** 1 Faculty of Health Sciences & Medicine, University Lucerne, Frohburgstrasse 3, 6002 Luzern, Switzerland; 2 Sarcoma Service, Department of Orthopedics and Trauma, Sarcoma Center, LUKS University Hospital, 6000 Luzern, Switzerland; 3 Sarcoma Service, Department of Radiation Oncology, Sarcoma Center, LUKS University Hospital, 6000 Luzern, Switzerland

**Keywords:** soft tissue sarcoma, ultra-hypofractionated preoperative radiotherapy (uhpRT), systemic therapy, PD-1 checkpoint inhibition, therapy de-intensification, survival outcomes, treatment toxicity, value-based healthcare, real-world evidence, high-risk sarcoma management

## Abstract

**Background/Objectives:** The SU2C-SARC032 randomized controlled trial (RCT) tested pembrolizumab combined with preoperative normofractionated radiotherapy as an intensified treatment for high-risk stage III resectable soft tissue sarcoma (STS), demonstrating a moderate improvement in disease-free survival (DFS) compared to preoperative radiotherapy alone, but accompanied by significantly increased toxicity, prolonged treatment durations, elevated resource source, and limited real-world applicability. To address the gap between highly controlled trial outcomes and routine clinical practice, this comparative analysis evaluated a streamlined ultra-hypofractionated preoperative radiotherapy (uhpRT) protocol using real-world data (RWD) as a potentially more balanced approach. **Methods:** Prospectively collected observational RWD from 54 consecutive patients with Stage III (T2 N0 M0) high-risk resectable STS treated at a single institution with uhpRT (25 Gy in 5 fractions in one week, no systemic therapy, median interval of 14 days to surgery) were analyzed. Survival endpoints (overall survival [OS], DFS, local disease-free survival [LDFS], distant disease-free survival [DDFS]), toxicity, and treatment duration were compared qualitatively with published outcomes from the SU2C-SARC032 trial’s intensified pembrolizumab arm and control arm. **Results:** At 2 years, the optimized uhpRT protocol achieved OS (90%), DFS (66%), and DDFS (70%) comparable to the intensified pembrolizumab arm (OS: 88%, DFS: 67%, DDFS (67%)) and clearly exceeded outcomes of the control arm (OS/DFS/DDFS: 85%/52%/52%). Importantly, the uhpRT protocol markedly reduced treatment-related toxicities (0% Grade 3/4 events vs. 56% in the intensified trial arm) and total treatment duration (<1 month vs. 3–11 months). **Conclusions:** These findings challenge the necessity of broad treatment intensification for high-risk localized STS, strongly supporting the concept of therapeutic optimization. Given substantial real-world variability in treatment practices and feasibility highlighted by recent research, our findings advocate for treatment strategies that prioritize realistic applicability, patient safety, and value-based care principles over pure intensification.

## Simple Summary

Soft tissue sarcomas (STS) are rare, aggressive cancers that require carefully balanced treatment strategies to improve survival without excessive side effects. Standard treatment for high-risk localized STS typically includes preoperative radiotherapy and surgery. Recent trials have explored intensifying treatment by adding immunotherapy (e.g., pembrolizumab), but these approaches often lead to increased toxicity, prolonged treatment times, and higher healthcare demands. In this study, we evaluated an optimized, short-course preoperative ultra-hypofractionated radiotherapy (uhpRT) using real-world data, highlighting differences between outcomes achieved under everyday clinical conditions versus highly standardized trial environments. Comparing our outcomes with those from a recent randomized controlled trial (SU2C-SARC032) that tested intensified therapy with pembrolizumab, our streamlined uhpRT protocol achieved similar survival outcomes while markedly reducing toxicity, treatment duration, and resource utilization. Our results underline the importance of therapeutic optimization, emphasizing efficacy, safety, and real-world applicability over routine intensification.

## 1. Introduction

Soft tissue sarcomas (STS) are rare mesenchymal tumors with diverse histological subtypes and anatomical locations, posing challenges in diagnosis, staging, and treatment [1]. In Switzerland, about 330 new cases per year are officially indicated by the National Registration of Cancers (Nationales Krebsregister der Schweiz) [2,3]. High-risk cases, such as Stage III (T2 N0 M0) high-grade sarcomas, are aggressive, with high recurrence and metastasis rates, leading to significant morbidity and mortality [4,5,6]. Effective STS management requires a multidisciplinary approach, with surgery as the cornerstone for localized disease. Preoperative radiotherapy is a standard adjunct to improve local control in high-risk tumors [7,8]. The role of currently available systemic therapies for non-metastatic STS is still unclear, as chemotherapies such as doxorubicin and ifosfamide offer, based on available limited data, only modest survival benefits and are associated with significant toxicity, thereby limiting their role in routine practice [9,10,11]. Although treatment intensification, for example via extended systemic therapy or immunotherapy, may appear attractive, these regimens often increase toxicity, treatment duration, and resource demands. Despite advances in multimodal strategies, balancing efficacy and safety—key tenets of value-based healthcare—remains an urgent priority, necessitating the pursuit of optimized (i.e., deintensified) treatment protocols and the generation of real-world evidence [12,13].

Furthermore, the reproducibility of outcomes reported in randomized controlled trials (RCTs) remains uncertain when translated to real-world clinical practice. A recent multicenter sarcoma ring trial highlighted substantial inter-institutional variability in therapy planning and delivery, even when standardized protocols were employed, underscoring the risks of directly extrapolating RCT outcomes to routine care without critical evaluation of real-world feasibility and implementation [14].

The current standard of care for high-risk STS includes surgery combined with preoperative radiotherapy, while recent systemic therapies in use are selectively employed by some centers due to their limited efficacy, toxicity, and resource demands, as supported by the limited body of reliable data [9,15,16,17]. Recent trials, such as the SU2C-SARC032 RCT, are exploring combining pembrolizumab, an immune checkpoint inhibitor, with radiotherapy [18,19,20,21]. To reliably answer whether such intensification benefits patients, big data is required. This multicenter trial addressed a very important innovative question using a straightforward 2-arm protocol to evaluate the implementation of immunotherapy in the curative therapy of STS. The experimental arm (*n* = 64) of the trial demonstrated a 15% improvement in disease-free survival (DFS) in Grade 2 and 3 tumors compared to the control arm (*n* = 63, *p* = 0.06), while overall survival (OS) remained comparable between the test and control arms [18]. Grade 3 or higher adverse events were observed in 56% of patients in the experimental arm, raising concerns about the feasibility of therapy intensification in routine practice. The trial’s stringent inclusion criteria limit its generalizability to broader real-world patient populations. In contrast, in real-world settings, high-risk STS management often involves patients with comorbidities, advanced age, larger tumors, or prior unplanned surgeries, and local recurrences—all conditions typically excluded from clinical trials.

Observational RWD studies complement RCTs by capturing the heterogeneity of real-world populations, offering inclusive assessments of treatment efficacy and safety, and addressing gaps in the trial data arena [22].

Against this backdrop, the present analysis compares real-world evidence from an ultra-hypofractionated preoperative radiotherapy (uhpRT) protocol for high-risk STS with the published SU2C-SARC032 RCT results. Emphasizing an approach aligned with “therapeutic optimization” over intensification, this study underscores the importance of minimizing treatment-related toxicity, shortening total treatment time, and preserving efficacy in a value-based healthcare context.

However, methodological concerns have emerged regarding recent intensified therapy trials, particularly the SU2C-SARC032 trial, which included Grade 2 tumors classified as stage III according to the outdated AJCC 7th edition (2009). According to the current AJCC 8th edition (2017), Grade 2 lesions are no longer classified as stage III. The inclusion of these lower-risk tumors likely skews survival outcomes, inflating the observed effectiveness of intensified systemic therapy. Therefore, real-world analyses that accurately reflect contemporary staging criteria and patient heterogeneity are critical for validating treatment strategies.

## 2. Materials and Methods

### 2.1. Study Design

This study analyzed prospectively collected data of a RWD uhpRT RWD cohort of consecutive high-risk STS patients treated at a single institution over a 4.5-year period [23]. The primary objective was to compare these RWD outcomes to the results of the SU2C-SARC032 RCT, a multicenter randomized controlled trial published in November 2024 in The Lancet [18]. The SU2C-SARC032 RCT was chosen as the benchmark for comparison due to its control arm using pre-operative radiation only, rendering it comparable to its own RWD protocol cohort, and due to its clinical relevance, rigorous methodology, and similar timeframe for patient recruitment. All included consecutive RWD patients meeting the same specific eligibility criteria as given in the SU2C-SARC032 trial; however, without exclusions based on age, comorbidities, tumor location, or prior interventions such as unplanned surgeries or previous irradiation. This inclusive approach ensured a representative real-world cohort reflective of the complexities encountered in clinical practice.

### 2.2. Patient Population

#### 2.2.1. uhpRT Cohort

The uhpRT RWD cohort included all consecutive patients who met the SU2C-SARC032 RCT conditions (resectable Stage III (T2 N0 M0) grade 2/3 STS as according to AJCC 7th edition) and were treated between April 2020 and November 2024 at a single institution. In contrast to the RCT benchmark trial, no exclusions were made based on age, comorbidities, tumor size, location, or prior interventions, resulting in a heterogeneous real-world population. This inclusiveness allowed for the evaluation of treatment efficacy across diverse clinical scenarios, including patients with prior unplanned excisions (“whoops” surgeries), reoperations for locally recurrent disease, and retroperitoneal or trunk wall lesions. Excluded were 13 patients treated with the same protocol in the same time interval; however, they were diagnosed with G1 lesions.

#### 2.2.2. SU2C-SARC032 RCT Cohort

The SU2C-SARC032 trial enrolled patients with resectable Stage III high-grade STS G2/3 that were required to meet specific fitness thresholds for pembrolizumab and radiotherapy. Exclusion criteria encompassed prior systemic therapy, significant comorbidities, and inadequate organ function. Additionally, patients with retroperitoneal tumors, prior unplanned surgeries, or residual disease were excluded, reflecting the trial’s selective design.

#### 2.2.3. Comparison of Inclusion Criteria

The broader inclusion criteria of the uhpRT RWD cohort allowed for the analysis of outcomes in a more heterogeneous population with respect to real-life practice than the controlled SU2C-SARC032 trial. This real-world approach enhances the generalizability of the findings and provides valuable insights into the protocol’s performance in less idealized clinical conditions (Table 1).

### 2.3. Treatment Protocols

The uhpRT RWD cohort followed a predefined streamlined protocol comprising ultra-hypofractionated preoperative radiotherapy, delivering a total of 25 Gy in 5 fractions over one week. Limb-salvage surgery was performed approximately two weeks (median 14 days) following radiotherapy completion. This treatment protocol deliberately avoided systemic therapy to minimize patient burden and enhance clinical feasibility and tolerability.

In contrast, the SU2C-SARC032 trial evaluated two distinct treatment arms:

- **Control Arm:** Patients received conventional normofractionated radiotherapy (50 Gy in 25 fractions over 5 weeks) followed by surgery without systemic therapy.
- **Experimental Arm:** Patients received conventional normofractionated radiotherapy combined with systemic therapy (pembrolizumab), administered over a median of 14 cycles (maximum 17), spanning approximately 3–11 months (Table 2).

### 2.4. Outcome Measures

#### 2.4.1. uhpRT Study Endpoints

The endpoints were chosen according to the SU2C-SARC032 Trial endpoints, in order to comparatively evaluate treatment effectiveness: OS, defined as the time from the start of radiotherapy to death from any cause; DFS, defined as the time from the start of radiotherapy to any recurrence (local, nodal, regional, or distant) or death; LDFS measuring the time to local recurrence, and DDFS measuring the time to distant metastasis. These endpoints were evaluated at two years, with survival probabilities estimated using Kaplan–Meier analysis. Follow-up time was calculated from the start of radiotherapy to the last documented clinical contact or event, and patients lost to follow-up were censored at their last recorded visit. This approach reflects real-world practice and allows for a pragmatic assessment of treatment outcomes.

#### 2.4.2. SU2C-SARC032 Trial Endpoints

In the SU2C-SARC032 trial, DFS was the primary endpoint, defined as the time from randomization to recurrence or death. Secondary endpoints included OS, LDFS, and DDFS, all measured using Kaplan–Meier analysis. The SU2C-SARC032 reached a longer follow-up duration, with a median of 43 months for both the experimental and control arms. This extended follow-up allowed for a more comprehensive assessment of long-term outcomes such as distant metastases and late recurrences.

#### 2.4.3. Alignment and Deviations

While the definitions of OS, DFS, LDFS, and DDFS were consistent across both studies, notable differences existed in follow-up duration and data collection. The uhpRT RWD cohort had a shorter median follow-up of only 30 months, limiting the evaluation of long-term outcomes, whereas the SU2C-SARC032 controlled trial design allowed for more structured follow-up over a median of 43 months. Additionally, the uhpRT study relied on real-world data, introducing variability in patient follow-up intervals, while the SU2C-SARC032 maintained a uniform follow-up schedule. These differences in methodology were carefully considered to ensure that comparisons between the uhpRT RWD cohort and the SU2C-SARC032 trial were kept as valid as possible.

### 2.5. Statistical Analysis

#### 2.5.1. Cohort Analysis

Kaplan–Meier survival curves were generated for OS, DFS, LDFS, and DDFS in the uhpRT cohort, with median survival times and 95% confidence intervals (CIs) reported. Cox proportional hazards models were used to assess associations between clinical variables, such as tumor grade, patient age, tumor size, and prior surgeries, and survival outcomes, providing insights into prognostic factors within a real-world population.

#### 2.5.2. Comparison with SU2C-SARC032 Trial

Published summary statistics from the SU2C-SARC032 trial were used for indirect comparisons. Kaplan–Meier survival probabilities and hazard ratios for both trial arms were extracted, and subgroup analyses by tumor grade, age, and tumor size were conducted to align the uhpRT RWD cohort with the SU2C-SARC032 study populations.

#### 2.5.3. Adjustments and Limitations

Due to the lack of access to raw data from the SU2C-SARC032 trial (which was only released 3 years after publication), propensity score matching was not feasible. Sensitivity analyses were conducted to account for known baseline differences, such as tumor size/location or prior interventions. According to the study’s Supplementary Materials in The Lancet paper, raw data from the SU2C-SARC032 trial will only be made available in three years, limiting the ability to perform direct comparisons in the current analysis. Despite these constraints, consistent endpoint definitions and Kaplan–Meier methodologies support meaningful qualitative comparisons, providing valuable insights into the effectiveness of the uhpRT protocol.

## 3. Results

### 3.1. Patient Characteristics and Treatment Protocols

This comparative analysis included 54 consecutive patients with Stage III (T2 N0 M0) STS treated at a single institution using the uhpRT protocol. Their outcomes were compared with those of 63 and 64 patients in the control and experimental arms, respectively, of the SU2C-SARC032 RCT, a multicenter randomized controlled trial conducted across 20 international institutions. The analysis focuses on survival outcomes, safety, and treatment feasibility, emphasizing differences in baseline characteristics, treatment protocols, and the real-world applicability of the uhpRT protocol compared to the controlled environment of the SU2C-SARC032 RCT (Table 3).

#### 3.1.1. Baseline Characteristics

The median follow-up in the uhpRT RWD cohort was 30.2 months, compared to 43 months in both arms of the SU2C-SARC032 trial. Patients in the uhpRT RWD cohort were slightly older, with a mean age of 63 years (range: 38–86), compared to 60 years and 59 years in the control and experimental arms, respectively. High-grade (Grade 3) tumors were more prevalent in the uhpRT RWD cohort (76%) compared to the trial cohorts (68% and 66%). The uhpRT RWD cohort exhibited greater histological diversity, with undifferentiated pleomorphic sarcoma (UPS) representing 28% of cases, compared to 83% in the SU2C-SARC032 trial arms. Median tumor volumes in the uhpRT RWD cohort measured 161 cm³, while volumetric data were not reported for the SU2C-SARC032 trial cohorts. Tumor sizes based on the longest diameter did not significantly differ across cohorts.

The uhpRT RWD cohort included real-world complexities not captured in the trial populations, such as prior unplanned excisions (“whoops” surgeries; 9%), reoperations for locally recurrent disease (11%), previous radiotherapy (5%), and retroperitoneal tumor location (9%). These factors reflect the broader inclusiveness of the uhpRT RWD cohort, which offers insights into the performance of the treatment protocol in less idealized clinical settings. This heterogeneity underscores the real-world adaptability and feasibility of the uhpRT protocol compared to the controlled trial environment.

#### 3.1.2. Treatment Protocols

The uhpRT RWD cohort adhered to the predefined ultra-hypofractionated regimen (25 Gy in 5 fractions over one week), followed by surgery at a median interval of 14 days [23]. Only one patient (2%) deviated from the protocol by receiving preoperative chemotherapy due to specific clinical indications. No postoperative chemotherapy was administered.

In comparison, the SU2C-SARC032 trial utilized conventional radiotherapy (50 Gy in 25 fractions over 5 weeks) and included pembrolizumab in its experimental arm, administered over 14–17 cycles (approximately 3–11 months). Surgical management was limb-sparing in all groups, with no amputations recorded.

### 3.2. Survival Outcomes

Survival outcomes of the uhpRT RWD cohort were evaluated and compared with the control and experimental arms of the SU2C-SARC032 trial to assess its effectiveness. Kaplan–Meier curves were generated for OS, DFS, LDFS, and DDFS at two years (Table 4).

#### 3.2.1. Overall Survival (OS)

**Figure 1:**
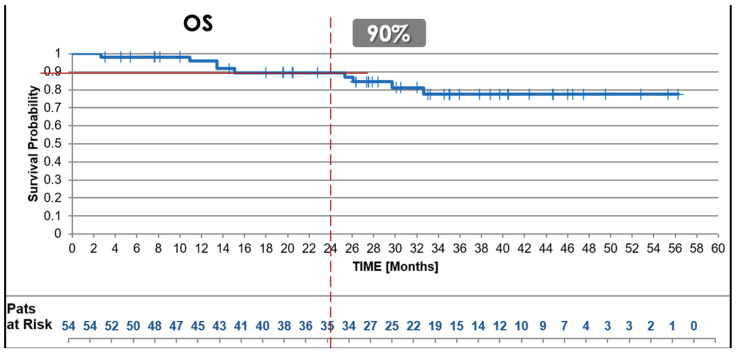
Overall Survival. Kaplan–Meier survival curves depicting 2-year Overall Survival (OS) in the uhpRT real-world cohort compared qualitatively to outcomes of the control and intensified pembrolizumab arms from the SU2C-SARC032 trial. The uhpRT protocol demonstrated comparable OS rates despite the shorter treatment duration and absence of systemic therapy.

The 2-year OS rate for the uhpRT RWD cohort was 90%, comparable to the experimental (88%) and the control arm (85%; *p* = 0.28, trial control vs. test arm) of the SU2C-SARC032 trial (Figure 1).

#### 3.2.2. Disease-Free Survival (DFS)

**Figure 2:**
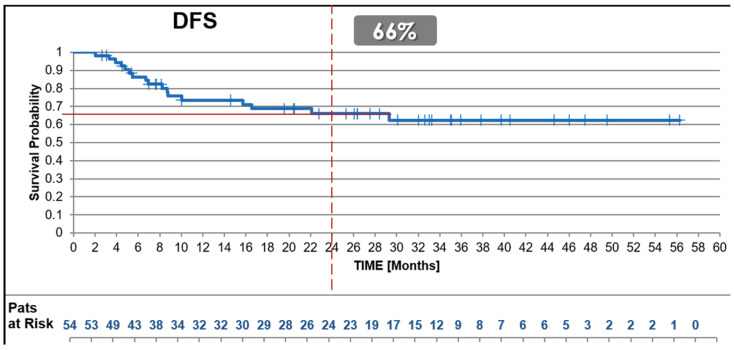
Disease-free Survival. Kaplan–Meier survival curves illustrating 2-year Disease-Free Survival (DFS) in patients treated with the uhpRT protocol compared with the control and intensified pembrolizumab arms of the SU2C-SARC032 trial. DFS rates in the uhpRT RWD cohort closely matched those of the intensified treatment arm and significantly exceeded those of the control arm.

The 2-year DFS rate for the uhpRT RWD cohort was 66%, compared to 67% observed in the experimental arm of the SU2C-SARC032 trial and 52% (*p* = 0.035, trial control vs. test arm) reported in the control arm (Figure 2).

#### 3.2.3. Local Disease-Free Survival (LDFS)

**Figure 3:**
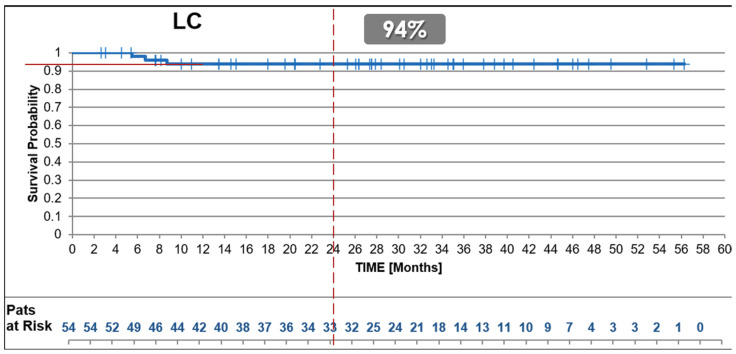
Local Disease-Free-Survival. Kaplan–Meier curves showing 2-year Local Disease-Free Survival (LDFS) outcomes for the uhpRT RWD cohort in comparison with published LDFS results from the SU2C-SARC032 trial. The uhpRT RWD cohort exhibited excellent local control rates, surpassing outcomes of both control and intensified treatment arms.

The uhpRT RWD cohort achieved a 2-year LDFS of 94%, higher than the approximately 85% reported in both the control and experimental arms of the SU2C-SARC032 trial, as according to Figure 3 in the article, while 100% for the control arm and ~96% (2 failures) for the permbrolizumab test arm as according to the description in the text (Figure 3).

#### 3.2.4. Distant Disease-Free Survival (DDFS)

**Figure 4:**
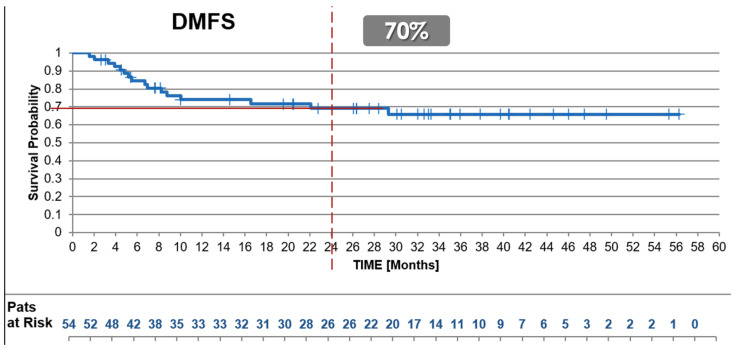
Distant-Disease-Free Survival. Kaplan–Meier survival curves presenting 2-year Distant Disease-Free Survival (DDFS) of the uhpRT RWD cohort versus the SU2C-SARC032 trial cohorts. The uhpRT protocol demonstrated comparable efficacy in preventing distant metastases, mirroring outcomes achieved with intensified pembrolizumab treatment and surpassing the control arm.

The 2-year DDFS for the uhpRT RWD cohort was 70%, closely matching the 67% observed in the experimental arm of the SU2C-SARC032 trial and surpassing the 52% (*p* = 0.08, trial control vs. test arm) reported in its control arm (Figure 4).

### 3.3. Safety and Adverse Events

The safety profile of the uhpRT protocol was limited, comparable to that of the SU2C-SARC032 trial, as in the RCT, no differentiation between early and late-term effects was indicated. The own evaluation included acute radiotherapy toxicity, late-term toxicity, and wound complication rate. The findings emphasized the markedly superior tolerability of the uhpRT protocol, particularly as it avoids the systemic toxicities associated with pembrolizumab (Table 5).

#### 3.3.1. Radiation-Related Early Side Effects

Early radiation-related side effects in the uhpRT RWD cohort were minimal. Most patients (96%) experienced no early skin reactions (Grade 0), while the remaining 4% exhibited only mild skin reactions (Grade 1). No cases of moderate or severe acute toxicity (Grade 2 or higher) were reported. Although detailed acute toxicity data were not explicitly published for the SU2C-SARC 032 trial, reported rates of severe adverse events (Grade 3/4) were high, with 31% in the control arm and 56% in the experimental arm. These findings highlight the superior short-term tolerability of the uhpRT protocol.

#### 3.3.2. Wound Complications

The uhpRT RWD cohort demonstrated a low wound complication rate of 12%. This result supports the feasibility of the ‘short interval’ surgical approach utilized in the uhpRT protocol due to the excellent early tolerance. Importantly, the shortened interval between the completion of radiotherapy and surgery (median 14 days) did not appear to compromise wound healing. While comparable data on wound complications from the SU2C-SARC032 trial were not explicitly reported, the observed rate in the uhpRT RWD cohort reflects a favorable balance between treatment efficiency and surgical outcomes.

#### 3.3.3. Late-Term Effects

Late-term effect data following the uhpRT RWD cohort revealed no instances of any severe (Grade 3 or 4) toxicity during the still short study period—as expected considering the relatively lower radio-biological effective dose of 5 × 5 Gy as compared to the normofractionated schedule in the RCT (: equivalent doses EqD2 for late responding normal tissues (a/b = 2 Gy): 44 Gy vs. 50 Gy). This contrasts with the experimental arm of the SU2C-SARC 032 trial, where 47% of patients experienced at least one serious adverse event, likely attributable to pembrolizumab. The absence of late-term severe toxicity so far in the uhpRT RWD cohort reinforces the protocol’s potential to minimize long-term treatment-related complications.

#### 3.3.4. Overall Adverse Events

The uhpRT RWD protocol exhibited a notably superior safety profile compared to the SU2C-SARC032 trial. None of the patients in the uhpRT RWD cohort experienced Grade 3/4 toxicities or serious adverse events during the study period. In contrast, the SU2C-SARC032 trial reported substantial toxicity, with 31% of patients in the control arm and 56% in the experimental arm experiencing Grade 3/4 adverse events. Additionally, at least one serious adverse event occurred in 19% and 47% of patients in the control and experimental arms, respectively. These findings underscore the efficacy of the uhpRT RWD protocol in achieving disease control while significantly reducing the risk of severe treatment-related complications, further supporting its applicability in real-world clinical settings.

### 3.4. Subgroup Analysis

Due to the modest sample size, all subgroup analyses presented below are exploratory, intended primarily for hypothesis generation and cautious interpretation. Statistical validation (e.g., *p*-values) was not performed, and the following observations should not be considered conclusive.

#### 3.4.1. Tumor Grade

Patients with Grade 2 tumors (*n* = 13) in the RWD cohort showed favorable survival outcomes, achieving a 2-year OS of 100% and DFS of 75%. In contrast, patients with Grade 3 tumors (*n* = 41) had slightly lower outcomes, with a 2-year OS of 86% and DFS of 55%. Although direct comparisons with the SU2C-SARC032 trial were not feasible due to unavailable subgroup data, these observations support tumor grade as a clinically relevant prognostic indicator.

#### 3.4.2. Patient Age

The uhpRT protocol demonstrated similarly effective outcomes across age groups, with younger patients (<65 years) showing a 2-year OS of 89% and older patients (≥65 years) 87%. DFS was comparably balanced, with rates of 63% for younger and 61% for older patients, indicating that age alone may not significantly influence outcomes within this treatment protocol.

#### 3.4.3. Tumor Volume

Patients with tumors larger than the median size (161 cm³) exhibited slightly reduced local (91% vs. 97%) and distant (67% vs. 77%) disease-free survival rates compared to smaller tumors. This observation aligns with clinical expectations—larger tumors typically correlate with higher metastatic risk—but must be interpreted cautiously given limited subgroup sizes, lack of statistical validation, and inherent selection bias (exclusion of metastatic cases at baseline). Future larger-scale studies are necessary to confirm these observations definitively.

## 4. Discussion

This comparative analysis demonstrates that our optimized uhpRT protocol, devoid of systemic therapy, achieves comparable short-to-intermediate-term survival outcomes to the intensified pembrolizumab regimen assessed in the SU2C-SARC032 randomized controlled trial (RCT). Our findings provide strong evidence that treatment optimization—characterized by shorter durations, substantially lower toxicity, and reduced healthcare resource consumption—may offer significant clinical and economic advantages over therapy intensification in high-risk localized soft tissue sarcomas.

The chosen 5-fraction ultra-hypofractionated regimen (25 Gy over 1 week) was based on existing evidence demonstrating comparable efficacy and improved acute tolerability relative to conventional 5-week normofractionated radiotherapy [23,24,25,26,27] (regarding [23,24]: please see also Table 1 and Table 3). Additionally, the shorter regimen potentially leverages favorable immunological effects typically associated with hypofractionated schedules (≤8 fractions), which may enhance local and systemic antitumor immune responses [28]. Indeed, larger fraction sizes have been reported to potentiate immunogenic cell death, highlighting a mechanistic basis for combining short-course radiotherapy with immunotherapeutic strategies if needed [29,30,31,32,33]. While the SU2C-SARC032 trial importantly addressed the innovative question regarding immunotherapy intensification, its preliminary results, though noteworthy for a ~15% absolute improvement in disease-free survival (DFS), revealed substantial increases in toxicity, prolonged treatment duration, and higher resource requirements. The reported DFS benefit was found statistically significant when summarizing G2 and G3 cases, while the G3 subgroup (high risk subgroup, as according to the 2017 AJCC 8th edition) itself was not found significantly different among the control and test arm (*p* = 0.06), G2 cases showed no differences (*p* = 0.78) (Table 4). Notably, the extended duration of pembrolizumab treatment (up to 17 cycles spanning nearly one year) in the SU2C-SARC032 trial introduces an additional potential bias. This prolonged systemic exposure could artificially delay recurrence, thus inflating disease-free survival rates without necessarily reflecting a genuine long-term therapeutic benefit. By comparison, our optimized uhpRT protocol, designed explicitly to minimize patient burden and duration of therapy, addresses this issue by demonstrating comparable disease control within a significantly shorter treatment timeframe. Specifically, 56% of patients in the intensified regimen experienced severe (Grade 3/4) toxicities, raising critical questions regarding its broader applicability and necessity, particularly in real-world clinical settings [34,35,36]. The median follow-up time in our uhpRT RWD cohort was 30 months, which is shorter than that reported in the SU2C-SARC032 trial (43 months). This limits the assessment of long-term outcomes, particularly late recurrences, distant metastases, and late toxicity. However, approximately 80% of soft tissue sarcoma recurrences—both local and distant—are known to occur within the first three years after treatment [37], suggesting that our findings provide a valid estimate of mid-term disease control [37].

These outcomes reflect broader concerns outlined by Braik et al. regarding the unclear benefit and potentially disproportionate risks associated with systemic therapy intensification in localized soft tissue sarcoma [9]. We acknowledge the recent CTOS consensus (September 2024), which advises against adopting hypofractionated preoperative radiotherapy outside of clinical trials [38]. However, our uhpRT program began in March 2020 and has, from the outset, been implemented within a structured real-world framework, with prospective documentation of toxicity and patient-reported outcomes. This approach supports responsible, data-driven adaptation of care models and may contribute complementary evidence toward future refinement of consensus guidelines. The recently proposed Common Sense Oncology principles further support this critical approach to clinical trial design and reporting, advocating for endpoints that directly translate into meaningful patient benefits, such as overall survival and health-related quality of life (HRQOL). These principles underline the necessity for clear communication regarding absolute clinical benefits, realistic toxicity profiles, and overall patient burden, thus reinforcing the importance of prioritizing treatment strategies that provide genuine value rather than purely statistical advantages [12,39].

In stark contrast, our uhpRT protocol exhibited no severe adverse events (Grade 3/4), highlighting substantial gains in patient safety and tolerability [23,24,38]. In contrast, the pembrolizumab arm of the SU2C-SARC032 trial reported severe (Grade 3/4) adverse events in 56% of patients, with nearly half experiencing serious adverse events (SAEs), significantly impacting patient quality of life. The observed low wound complication rate (12%) is consistent with our previously published experience in normofractionated preoperative RT and likely reflects both meticulous surgical technique and radiation planning [40]. Notably, PTVs were manually edited to minimize exposure of skin, bone, and joints, and underwent systematic double-review. The long-term collaboration of the same surgical-radiation team (>20 years) may have further contributed to the consistent mitigation of early complications. Importantly, no radiation-induced bone fractures have occurred to date, likely due to strict avoidance of circumferential or extended bone exposure during PTV delineation. Based on estimated α/β ratios of 2 for late-responding tissues and ~4 for sarcoma tumor control, the 5 × 5 Gy regimen corresponds to an EqD2 of approximately 44 Gy and 37.5 Gy, respectively. The BED for 5 × 5.0 Gy of ~56 Gy is somewhat lower than that for 25 × 2.0 Gy with ~66 Gy, including the time correction. The substantially shorter total treatment time (1 vs. 5 weeks) compensates at least in part for the lower BED. Our observed 2-year LC rate of 94% underscores the efficacy of this protocol when implemented with rigorous QA and multidisciplinary coordination. However, in the comparator trial, quality-of-life data were not comprehensively reported. These toxicity findings highlight the critical importance of balancing survival outcomes against treatment-related morbidity, further underscoring the advantages of a safer and more patient-centered approach, such as our uhpRT protocol. These findings underscore the clinical relevance of optimizing locoregional strategies such as radiotherapy, while recognizing that innovations in systemic therapy for STS are urgently needed and must be developed through rational, patient-centered trial designs, as highlighted in the broader Common Sense Oncology framework [12,13]. By significantly shortening total treatment duration to less than one month, our approach also addresses practical limitations inherent to intensified regimens, such as prolonged hospital stays, increased patient burden, reduced compliance, and greater financial toxicity. Adopting optimized regimens, such as uhpRT, could significantly decrease both direct and indirect healthcare costs, thus aligning with global efforts to implement sustainable, value-based healthcare practices [41,42,43].

Furthermore, the inclusivity of our real-world data cohort, comprising patients with comorbidities, recurrences, prior interventions, and diverse anatomical tumor sites, reinforces the generalizability and real-world relevance of our findings [44,45,46,47,48]. However, this patient heterogeneity may impact outcome comparisons with more selectively enrolled trial populations. Indeed, extrapolating the results from highly controlled clinical trial settings to routine practice without consideration of real-world variability is problematic, as demonstrated by the sarcoma ring trial [14]. This trial revealed significant discrepancies between institutions in treatment planning, despite uniform guidelines. Therefore, it is imperative to not only compare outcomes between therapeutic strategies but also to document and evaluate the exact treatments delivered across institutions. Without capturing these variances, outcome comparisons may equate fundamentally different practices, thus ‘comparing apples to oranges’ rather than providing a meaningful basis for clinical decision-making [39]. Additionally, the shorter median follow-up of 30 months in our cohort, compared to 43 months in the SU2C-SARC032 trial, may limit full assessment of late toxicities and long-term control. This limitation underscores the urgent need for international collaborative research networks specifically designed to improve accrual rates, enhance patient enrollment efficiency, and facilitate rapid generation of statistically robust evidence [49,50,51,52]. Our analysis thus provides valuable insights into treatment efficacy and feasibility within less idealized, everyday clinical scenarios. This approach aligns strongly with value-based healthcare principles, prioritizing patient outcomes, reducing treatment burden, and enhancing healthcare sustainability and equity [41,42,43].

Future investigations could further delineate the potential immunologic synergy of ultra-hypofractionated radiotherapy. Integrating immunotherapy with short-course RT in prospective studies may clarify whether such combinations can enhance disease control without incurring excessive toxicity. Additional research should also investigate the economic implications and long-term sustainability of optimized regimens, driving clinical practice toward evidence-based, patient-centered, and economically responsible sarcoma care. Including comprehensive patient-reported outcome measures (PROMs) alongside standard clinical endpoints will be essential to capture the broader impact of therapy on quality of life, functional recovery, and patient satisfaction [53].

## 5. Conclusions

Our real-world comparative analysis demonstrates that an optimized ultra-hypofractionated preoperative radiotherapy (uhpRT) protocol achieves short-to-intermediate-term survival outcomes comparable to the intensified pembrolizumab regimen explored in the SU2C-SARC032 phase III trial, but with substantially reduced toxicity, treatment duration, and resource demands.

These findings critically challenge the routine use of intensified systemic immunotherapy for high-risk localized soft tissue sarcomas, suggesting that the modest improvement in disease-free survival reported in the trial, combined with significantly increased toxicity, does not yet justify clinical adoption at this time. Instead, our results strongly support optimized, patient-centered treatment strategies consistent with value-based healthcare principles, highlighting the uhpRT approach’s efficiency, reduced toxicity, and potential immunological advantages.

Given the persistent high risk of distant metastases, further well-powered international trials remain essential to clearly define subgroups who might benefit from systemic immunotherapy. Moreover, as underscored by the sarcoma ring trial findings, future studies must rigorously document treatment delivery variability in real-world practice to ensure meaningful comparisons between therapeutic interventions. Adhering to Common Sense Oncology principles will further enhance the clinical relevance and applicability of future trial results, ultimately guiding oncology practice towards patient-centered, sustainable, and evidence-based care. Until stronger evidence emerges, intensified systemic treatments should remain cautiously and selectively applied based on individualized risk-benefit assessments. Furthermore, despite the observed improvement in disease-free survival in the pembrolizumab trial, no corresponding benefit in overall survival (OS) was demonstrated (88% pembrolizumab vs. 85% control, *p* = 0.28). Given the substantial increase in toxicity without OS benefit, the routine adoption of pembrolizumab in this setting remains unjustified outside clinical trials.

## Figures and Tables

**Table 1:** Comparison of Patient Cohorts.

Criteria	uhpRT RWD Cohort [23]	SU2C-SARC032 RCT
Stage	Stage III (T2 N0 M0)	Stage III (T2 N0 M0) *
Tumor Grade	Grade 2/3	Grade 2/3
Age restrictions	None	Specific thresholds for fitness
Comorbidities	No exclusions	Excluded significant comorbidities
Tumor Locations	Includes retroperitoneal or trunk wall	Excluded retroperitoneal
Prior Surgeries	Included unplanned surgeries or recurred disease	Excluded prior unplanned surgeries and recurred disease
Systemic Therapy History	No exclusions	Excluded prior systemic therapy
Residual Disease	included	excluded
Organ Function	No exclusions	Excluded patients with inadequate organ function

* The SARC032 trial used the 7th edition of AJCC from 2009 (and not the most current one), reason why the same criteria herein are also used for the uhpRT cohort.

**Table 2:** Comparison of Treatment Protocols [23].

Component	uhpRT RWD Cohort	SU2C-SARC032 RCT Control Arm	SU2C-SARC032 RCT Test Arm
Radiotherapy	25 Gy in 5 fractions (1 week)	50 Gy in 25 fractions (5 weeks)	50 Gy in 25 fractions (5 weeks)
Interval (RT-Surgery)	Median 14 days	Median 34 days	Median 36 days
Surgery	Limb-salvage only (100%)	Limb-salvage only (100%)	Limb-salvage only (100%)
Systemic Therapy	none	none	Pembrolizumab: median 14 cycles (max 17)
Toral treatment Duration	<1 month	2.5 months	3–11 months

**Table 3:** Comparison of treatment protocols.

Parameter	uhpRT RWD Cohort [23]	SU2C-SARC032 RCT Control Arm	SU2C-SARC032 RCT Test Arm
**Recruitment**	04.2020–11.2024 (4.5 y)	11.2017–11.2023 (6 y)	11.2017–11.2023 (6 y)
Interval Center Study Setup	Single institution	20 international institutions	20 international institutions
	Prospective RWD	Prospective RCT	Prospective RCT
N of patients	54	63	64
Follow-up, median (months)	30	43	43
**Sex**			
Female	21 (39%)	24 (38%)	23 (36%)
Male	33 (61%)	39 (62%)	41 (64%)
**Age**	63 (38–86)	60 (26–84)	59 (35–87)
**Ethnicity** (white)	100%	95%	80%
**Stage ^1^**	III	III	III
**TNM**			
T2-4 N0 M0	100%	100%	100%
rT2-4 N0 M0	6 (11%)	0	0
**Grade**			
Grade 2	13 (24%)	20 (32%)	22 (34%)
Grade 3	41 (76%)	43 (68%)	42 (66%)
**Histologic Subtype**			
Undiff. pleomorphic Sarcoma (UPS)	15 (28%)	48 (76%)	53 (83%)
Dedifferentiated Liposarcoma (DDLS)	12 (22%)	4 (6%)	4 (6%)
Myxofibrosarcoma (MFS)	12 (22%)	6 (10%)	7 (11%)
Undiff./Unclassified Sarcoma (NOS)	0	5 (8%)	0
Myxoid Liposarcoma (MLS)	5 (9%)	0	0
Leiomyosarcoma (LMS)	4 (7.5%)	0	0
Others	3 (5.5%)	0	0
Tumor Volume, median cc (range)	161 (18–3084)	NA	NA
Tumor Size (median cm, range)	10 (7–18)	10 (7–13)	11 (8–14)
Not available	5 (whoops)	NA	NA
**Tumor Location**			
Lower limb	25 (46%)	38 (60%)	41 (64%)
Lower limb girdle	6 (11%)	6 (10%)	3 (5%)
Upper limb	7 (13%)	9 (14%)	10 (16%)
Upper limb girdle	5 (9%)	10 (16%)	10 (16%)
Retroperitoneal	5 (9%)	0	0
Trunk wall	5 (9%)	0	0
others	1 (2%)	0	0
**Previously treated**			
Unpanned excision (whoops)	5 (9%)	0	0
Surgery for initial manifestation	6 (11%)	0	0
Previous adj. Radiation Therapy	3 (5.5%)	0	0
**Radiation Therapy**			
(Ultra-)hypofractionation	54 (100%)	2 (3%)	1 (2%)
normofractionation	0	61 (97%)	63 (98%)
**Systemic Therapy**			
Preoperative Chemotherapy	1 (2%)	0	0
Postoperative Chemotherapy	0	0	0
Pembrolizumab	0	0	100%
(PD-1-ihibitor, max 17, 3-weekly)			(median 14 cycles)
**Surgery**			
No surgery	0	1	2
Limb salvage	43/43 (100%)	NA	NA
Resection status (R0/1/2)	49 (91%)/5 (9%)/0	NA	NA
amputation	0	NA	NA

^1^ based on AJCC 7th edition from 2009, for comparison purposes.

**Table 4:** Details on Disease Control.

Parameter	RWD Cohort	SU2C-SARC032 Control Arm	SU2C-SARC032 Test Arm	*p*-Value RCT Arms
**Local Disease-free Survival @ 2y, all**	**94%**	**100%**	**97%**	
G2 (*n*)	100% (13)	NA	NA	X
G3 (*n*)	92% (41)	NA	NA	X
Female (*n* = 21)/male (*n* = 33)	94%/93%	NA	NA	X
Age ≤ 65 y/>65 y (*n* = 29/25)	92%/95%	NA	NA	X
**Distant Disease-free Survival @ 2y, all**	**70%**	**52%**	**67%**	
G2 (*n*)	80% (13)	NA	NA	X
G3 (*n*)	64% (41)	NA	NA	X
Female/male (*n*)	68% (21)/68% (33)	NA	NA	X
Age ≤ 65 y/>65 y (*n*)	58% (29)/68% (25)	NA	NA	X
**Disease-free Survival @ 2y, all**	**66%**	52%	**67%**	0.035
G2 (*n*)	80% (13)	74% (20)	80% (22)	0.78
G3 (*n*)	60% (41)	41% (43)	60% (42)	0.06
Female/male (*n*)	63% (21)/61% (33)	NA	NA	X
Age ≤ 65 y/>65 y (*n*)	54% (29)/72% (25)	NA	NA	X
**Overall Survival @ 2y, all**	**90%**	**85%**	**88%**	
G2 (*n*)	100% (13)	NA	NA	X
G3 (*n*)	86% (41)	NA	NA	X
Female/male (*n*)	94% (21)/86% (33)	NA	NA	X
Age ≤ 65 y/>65 y (*n*)	88% (29)/91% (25)	NA	NA	X

**Table 5:** Summary of safety outcomes.

Parameter	RWD Cohort	SU2C-SARC032 Control Arm	SU2C-SARC032 Test Arm	*p*-Value RCT
**Treatment Times**				
Radiation Therapy, weeks	1	5	5	X
End of Radiation Therapy to Surgery (median days)	14	34	36	X
Pembrolizumab, median cycles	0	0	14	X
(max 17, 3-weekly)				
Total Treatment Time, months (TTT)	<1	2.5	3–11	X
**Acute RT Toxicity**				
G0	52 (96%)	NA	NA	X
G1	2 (4%)	NA	NA	X
G2	0	NA	NA	X
**Wound Complication Rate**				
(acc. CAN-NCIC-SR2 trial)	12%	NA	NA	X
**Late-term Toxicity**				
G3/4	0	21 (31%)	39 (56%)	X
At least 1 serious adverse event	0	13 (19%)	31 (47%)	X

## Data Availability

The data presented in this study are available on request from the corresponding author.
